# Once-Weekly Hypofractionated Radiotherapy for Breast Cancer in Elderly Patients: Efficacy and Tolerance in 486 Patients

**DOI:** 10.1155/2018/8321871

**Published:** 2018-03-15

**Authors:** Javier Sanz, Min Zhao, Nuria Rodríguez, Raquel Granado, Palmira Foro, Ana Reig, Ismael Membrive, Manuel Algara

**Affiliations:** ^1^Radiation Oncology Department, Hospital del Mar, Parc de Salut Mar, Barcelona, Spain; ^2^Pompeu Fabra University, Barcelona, Spain; ^3^Autonomous University of Barcelona, Barcelona, Spain

## Abstract

**Purpose:**

Radiation therapy is a key treatment of breast cancer. Elderly patients with associated diseases that modify their performance status do not tolerate long periods of daily irradiation. The objective of this study is to analyze the results of weekly hypofractionated treatment in these patients.

**Material and Methods:**

Between 1992 and 2016, we included 486 elderly patients presenting concomitant pathology or sociofamilial problems in which it was not feasible to propose conventional treatment. They were treated with conservative surgery or mastectomy and then adjuvant hypofractionated irradiation, administering 5 Gy or 6.25 Gy in 6 fractions, once a week (total dose 30–37.5 Gy) over 6 weeks.

**Results:**

Breast cancer overall survival according to the Kaplan-Meier method at 5 years was 74.2% ± 2.3%; breast cancer disease-free survival was 90% ± 1.6%; local relapse-free survival was 96.5% ± 1% showing that patients die more from other causes and not from their neoplasia. Acute dermatitis was mild (75.6% of the patients grades I–III) and 30.6% had moderate chronic fibrosis.

**Conclusions:**

The once-weekly hypofractionated radiotherapy is a feasible and convenient option for elderly patients with breast cancer. It is a safe treatment modality with similar survival and local control results compared to standard fractionation, while the side effects are acceptable.

## 1. Introduction

Breast cancer is the most common tumor in women [1]. The incidence rate continues to increase slowly in both developed and underdeveloped countries. It is the leading cause of cancer mortality in women. Survival of breast cancer has really improved in the last 20 years, approximately 1.4% annually [2], mainly due to the early detection of this neoplasm, improvements in surgical techniques, systemic treatments, and radiotherapy.

The treatment of breast neoplasia combines surgery, systemic treatment, and radiotherapy. The most common regimen for the complementary treatment of breast cancer after conservative [3] or radical surgery has been the standard fractionation, consisting of 45–50 Gy in 25 fractions of 1.8 or 2 Gy/day, 5 days a week, for 33 days [4, 5]. These studies show that the incidence of local recurrence is significantly lower in the group of patients treated with radiotherapy, about an 8%–10% with respect to the nonirradiated ones in which the local recurrence reached 25%–35%, independently of other associated factors. One of the most important risk factors for the development of breast cancer is age [6]. Most cases are diagnosed between the ages of 35 and 80, nearly half of them diagnosed at the age of 70 or more years [7]. Currently, approximately 30% of patients in a radiotherapy department have breast cancer [8]. Elderly patients are frequently associated with concomitant diseases, cognitive impairment, sociofamilial problems, or functional limitation [9]. Patients often live far from the hospital and have difficulty to attend the radiotherapy sessions. Therefore, according to established practice, it is not always feasible for elderly patients to indicate a conventional, daily treatment [10]. This may lead to individuals avoiding complementary treatment after conservative surgery, or unnecessary abuse of radical mastectomy in the initial stages, which results in an undertreatment or overtreatment in a large number of cases. Therefore, it is advisable to plan a more convenient treatment regimen for the elderly patients while a similar local control result can be achieved.

Today, although the current standard is moderate daily hypofractionation, there are still many patients who cannot tolerate it and require a more adapted treatment to their condition. There are other fractionation schedules that can shorten the radiation treatment, demonstrating the same efficacy, without increasing complications. Aside improvement in quality of life [11], also short treatments can relieve the pressure of medical resources and logistical problems of access to the radiotherapy. One of the current challenges is the definition of irradiation schedules that are more comfortable for patients and easier to integrate with systemic therapies. In any case, there is no clear consensus on what should be used on a regular basis [12, 13].

The purpose of the present observational study is to analyze the results of weekly hypofractionated irradiation treatment in older patients with breast cancer in terms of survival, locoregional control, tolerance, and acute and chronic toxicity.

## 2. Materials and Methods

### 2.1. Patient Selection

Elderly patients with nonmetastatic breast neoplasia treated between 1992 and 2016 have been included. Inclusion criteria have been patients older than 70 years and/or those patients who do not tolerate daily travel to our radiotherapy department for several reasons such as those associated with concomitant diseases, cognitive impairment (according to the global deterioration scale or GDS of 4 or higher), sociofamilial problems, functional limitation, or living away from the treatment center. The criteria for the choice of initial surgery were the same as those used for young patients. So patients with I-II stages underwent conservative treatment and lymphadenectomy (or sentinel node selective biopsy), and patients with stage III were submitted to radical treatment with mastectomy and lymphadenectomy. In patients with conservative surgery, the margins were considered positive if the tumor reached the ink of the sample or close when they were negative but inferior to 3 mm. The characteristics of the patients are shown in Table 1.

### 2.2. Treatment

They received weekly hypofractionated irradiation within 2 months after surgery. The patients were treated with a cobalt unit or a linear accelerator by means 3D conformal tangential beams to the breast volume or field in field modulated techniques to optimize dosimetric distribution. All patients were treated in supine position, with abduction of ipsilateral arm utilizing an alpha-cradle immobilization.

The radiation schedule consisted of 6.25 Gy per fraction once a week but in the recent part of the series the fraction was reduced to 5 Gy with the aim of reducing the risk of secondary effects. Patients treated with conservative surgery or only biopsy received irradiation to the breast and mastectomized patients to the chest wall. In patients with T4 tumors treated with electrons to the chest wall a bolus was utilized. Patients with positive surgical margin were considered to receive radiotherapy to the tumor bed by adding 1 or 2 additional weekly doses at this level. The boost volume consisted in the tumor bed marked with clips and additional margin of 15 mm in all directions to obtain the PTV. The supraclavicular fossa was irradiated as a function of the nodal involvement, always in N2 cases and in patients with N1 involvement treated in the period from 2008 to 2016 according to our guideline and evidence published data [14]. In these cases, additional anterior and posterior fields to supraclavicular fossa and lymph node level III were utilized.

### 2.3. Evaluation and Follow-Up

All patients were evaluated weekly after the session for tolerance and underwent control of acute and chronic local toxicity according to the RTOG scale or the Common Terminology Criteria for Adverse Events v4.0 (CTCAE), indicating supportive treatment when necessary. Patients were followed periodically after the end of treatment, at a month, 6 months, and subsequently at least once a year.

Patients were included following the ethical principles for medical research according to Helsinki declaration and an informed consent was obtained from all the patients.

### 2.4. Statistical Analysis

The statistical analysis was performed with SPSS v22 (IBM SPSS, Chicago, IL). Chi-square and exact Fisher tests were employed to evaluate categorical variables. The actuarial rates of overall survival, disease-free survival, local relapse-free survival, and metastasis-free survival were calculated according to the Kaplan-Meier method and comparisons were made using the log-rank test.

## 3. Results

### 3.1. Patients and Treatment Characteristics

Four hundred and eighty-six patients have been included. The median age of patients was 79 ± 6.5 years (range 58–97). Half of the patients lived outside Barcelona, which makes it more difficult for them to get to the hospital. Breast conservative surgery was performed in 78.6% (73.3% tumorectomy and 5.3% quadrantectomy) and total mastectomy in 20% and only biopsy in 0.6% were performed by associated pathology that contraindicated the intervention or occasionally because of the refusal of the patient to be operated on. Patients without chemotherapy were treated postoperatively at 2.2 months (range: 1–3 months), and patients with adjuvant chemotherapy were treated 4.5 months (range: 2.5–4.6 months) after surgery. Hormonal therapy was utilized in 78.7% of the cases; among them 57.8% were treated with aromatase inhibitors. In 13.4% of the patients neoadjuvant or adjuvant chemotherapy was administered, mainly anthracyclines, taxanes, or CMF schedules.

Patients were treated in 13.4% of cases using 1.25 MV photons of a Cobalt-60 unit or by means of a linear accelerator unit so that 69.3% of the cases were treated with 6 MV photons (337 patients), and 17.3% of the cases corresponding to mastectomized patients were treated with electrons (84 patients). 80.7% of cases received a total of 6 sessions. 90.7% of patients received a fractionation of 6.25 Gy/fraction and 9.3% (45 patients) at 5 Gy/fraction up to a total dose of 30 Gy. An amount of 17.5% of patients with positive surgical margin after conservative surgery received a boost to the tumor bed of 1 or 2 additional doses (7.2% and 10.3% of patients, resp.). None of the patients treated with mastectomy received a boost. In 15% of the series, the patients received irradiation to the supraclavicular fosa and axillar level III cause of positivity of lymph nodes in the lymphadenectomy. Only 9 patients (1.8%) did not complete the treatment (1 patient received 4 sessions and 8 patients received 5 sessions) mainly due to complications of their comorbidities.

### 3.2. Recurrence and Survival

Local control was excellent, reaching 96.5% ± 1% at 5 years, and only 16 patients had local recurrence. After a median follow-up of 51 months (range: 1–163 months), the status of the patients was as follows: 341 (70%) were disease-free, 45 (9.3%) died as a result of cancer progression, and 95 (19.5%) died of another disease. Mortality was more frequently caused by concomitant diseases than by neoplasia.

**Figure 1:**
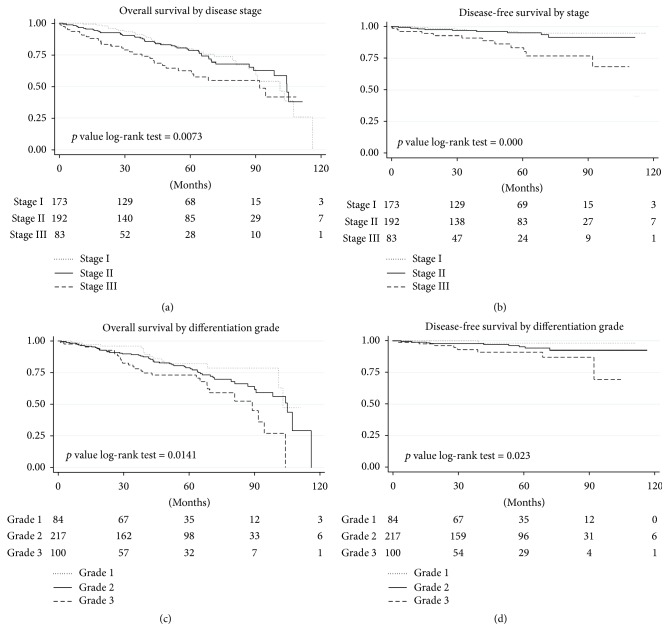
Overall and disease-free survival curves according to stage (a and b) and according to histologic grade (c and d).

**Figure 2:**
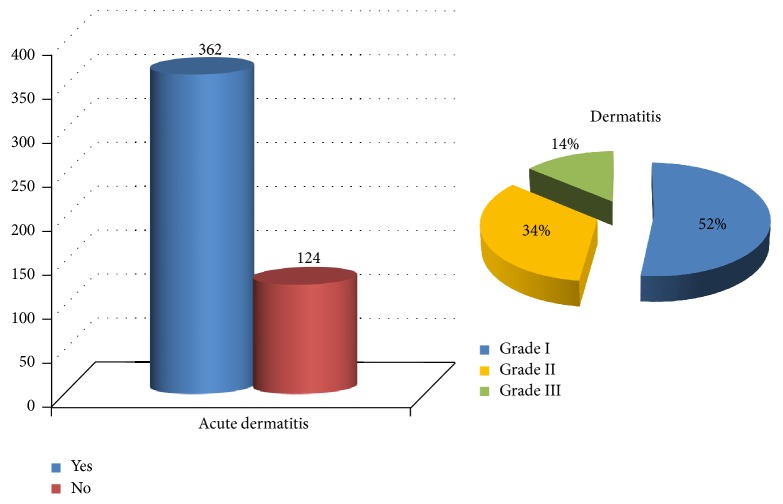
Acute toxicity and dermatitis grade according to RTOG criteria.

So, overall survival according to the Kaplan-Meier method at 5 years was 74.2% ± 2.3%; breast cancer specific survival was 90% ± 1.6%; relapse-free survival was 96.5% ± 1%; metastasis-free survival was 90% ± 1.6% (Figure 1). Estimated survival at 5 years according to the disease stage is presented in Figure 2: Patients with grade 1-2 histology had better survival rates than those of grade 3 (*p* = 0.002). Survival according to the histologic grade at 5 years is shown in Figure 2.

Survival according to the positivity of estrogen receptors was also significant. So, patients who had positive estrogen receptors have better survival than those whose receptors were negative. Overall, survival according to progesterone receptor is not statistically significant.

### 3.3. Treatment Tolerance and Toxicities

**Figure 3:**
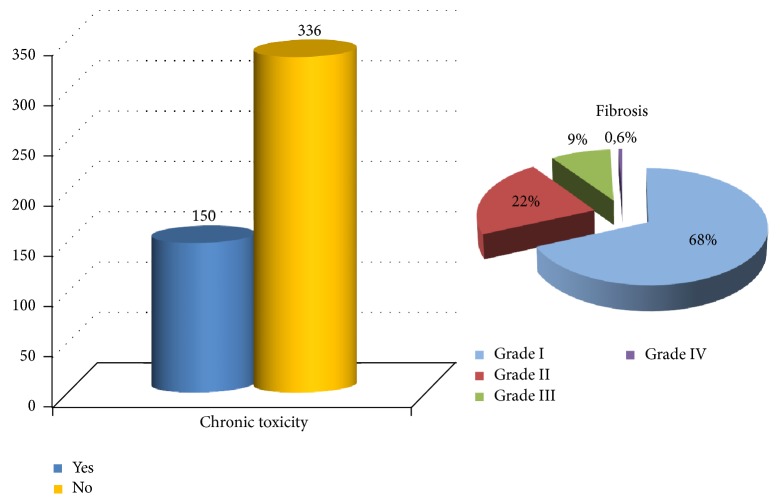
Chronic toxicity (any type) and fibrosis grade rates according to CTCAE v4.0 criteria.

The acute toxicity is presented in Figure 2. Among patients who have presented dermatitis, half of them were grade 1. Usually, the cases of acute dermatitis were resolved in 1-2 months with the appropriate topical moisturizing treatment for patients who have presented dermatitis grades 1 and 2 and with sulphadiazin cream for patients with grade 3. There were no differences in skin toxicity according to the treatment unit or the techniques utilized for irradiation (*p* = 0.092 and *p* = 0.174 for acute and chronic toxicity, resp.). When comparing acute toxicity among the fraction utilized, patients with 5 Gy per session presented dermatitis grade 1 in 65%, grade 2 in 30%, and grade 3 in 5%, and the patients treated with 6.25 Gy presented dermatitis grades 1, 2, or 3 in 47%, 32.6%, and 13.7%, respectively. In terms of chronic toxicity, 30.9% of the patients presented skin impairment (Figure 3). Among them, 88% presented fibrosis, 8.7% had hyperpigmentation or telangiectasia, and 3.3% had edema or mastitis. Among patients with fibrosis, only one patient was grade IV. The reporting of symptoms related to breast fibrosis was very unusual. Chronic toxicity was lower in patient treated with 5 Gy per session compared to patients treated with 6.25 Gy per session schedule (22.9% and 31.4%, resp.; *p* = 0.34). There were no cases of pulmonary or thoracic sequelae in terms of pneumonitis or rib fracture.

## 4. Discussion 

Complementary radiotherapy is classically indicated for patients who have undergone conservative surgery for all types of infiltrating tumors and many intraductal carcinoma tumors. The volume to be treated includes the entire breast with a total dose between 45 and 50 Gy, at a daily dose of 1.8–2 Gy as a classical or standard fractionation. The good results obtained with the conservative treatment provoke a new attempt to reduce the therapeutic aggressiveness, in this case focused on the radiotherapy. Hypofractionation programs are being developed in order to reduce the number of sessions, so as to increase patients' comfort, especially the comfort of elderly patients.

There is abundant experience in the hypofractionated treatment in patients with breast cancer (Table 2). Shelley et al. [15] demonstrated in a phase II study with 294 patients that treatment based on 40 Gy in 16 fractions of 2.5 Gy and in 22 days after a mean follow-up of 5.5 years achieved a relapse-free survival of 96.5% which had no difference in acute or chronic local toxicity. In 2010, Whelan demonstrated in his study with more than 1200 patients receiving 50 Gy in 25 fractions or 42.56 Gy in 16 fractions, after a 10-year follow-up, that there were no significant differences in local recurrence (6.7% versus 6.2%) and good aesthetic results were achieved (71.3% versus 69.8%, resp.). Acute toxicity of grade 3 or higher was only present in 3% in each group [11]. In the START-A trial, a regimen of 50 Gy in 25 fractions over 5 weeks was compared with 41.6 Gy or 39 Gy in 13 fractions over 5 weeks [16]. In START-B trial, a regimen of 50 Gy in 25 fractions over 5 weeks was compared with 40 Gy in 15 fractions over 3 weeks [17]. After approximately 10 years of follow-up, it has been confirmed that moderately daily hypofractionated irradiation is safe and effective for patients with early breast cancer. These results support the use of 40 Gy in 15 fractions (3 weeks), which has already been adopted by most centers as the standard therapy for women who require adjuvant radiotherapy for early stage invasive breast cancer.

More recently, results of the UK IMPORT LOW trial have been published. In this trial the patients were randomized to receive whole breast irradiation at a dose of 40 Gy in 15 fractions, 36 Gy to whole breast and 40 Gy to the partial breast (reduced-dose group) or 40 Gy to the partial breast only. This trail has showed noninferiority of partial breast and reduced-dose radiotherapy compared with the standard whole breast hypofractionated irradiation [18]. Also the same research group is investigating, in the HIGH IMPORT Trial, the intensification of hypofractionated treatments by comparing the whole breast irradiation of 40 Gy plus a sequential boost of 16 Gy (control group) with two test arms: arm 1 consisting in whole breast irradiation up to 36 Gy, the index quadrant up to 40 Gy, and concomitant boost up to 48 Gy in 15 fractions, or arm 2 with whole breast receiving 36 Gy, the index quadrant receiving 40 Gy, and a concomitant boost up to 53 Gy, also in 15 fractions. The trial is under recruitment.

From the radiobiological point of view, the limits to avoid increase in chronic toxicity seem to be about 3.2–3.3 Gy per fraction as reported by Qi et al. [19].

Although the 3-week daily schedule is a significant improvement compared to the traditional 5-6-week schedule, the requirements of daily therapy continue to be problem in regions with limited oncology care resources and in some cases the patients are excluded to receive appropriate postoperative treatment [20]. Favorable results from the randomized trials of the hypofractionated schedules described above have provided enough data to allow further investigation towards still more concentrated hypofractionated schedules with fewer sessions.

The first published clinical trial of once-weekly hypofractionated radiotherapy regimen was carried out in 1987 and published by Rostom et al. [21]. It was a phase II trial that applied a dose per session of 6.5 Gy/day, one session per week to older patients. It demonstrated a good locoregional control result and an acceptable cosmetic outcome. In 2006, Ortholan et al. published a similar study that included 150 patients undergoing both conservative and radical surgical treatment, using the same weekly hypofractionation schedule, in which a good clinical outcome was also demonstrated [22]. Other similar published studies have also shown similar locoregional control rates, with a slightly higher but acceptable chronic toxicity in most series [23–25]. More recently Dragun et al. have published a phase 2 study which included 42 patients who underwent conservative surgery and received weekly hypofractionated irradiation at 6 Gy/week in 5 weeks, total dose at 30 Gy, demonstrating the feasibility of the weekly schedule. At the same time, it was cost effective and had low acute toxicity rates, although further monitoring is required to confirm long-term results with a possible reduction of chronic toxicity according to dose reduction per fraction [26]. These results have been also confirmed in another series of Rovea et al. where 291 patients received 30 or 32.5 Gy in 5 fractions, with mild chronic toxicity in terms of fibrosis, edema, or hyperpigmentation and also achieving good or excellent cosmetic result in 86.4% of cases [27].

A common criticism of these studies regards their limited applicability in that they include mainly lymph-node-negative, postmenopausal patients with biologically favorable early stage invasive disease [28]. In the present study, we included 486 patients, the majority in early stage, who have received 5 Gy or 6.25 Gy in 6 sessions with 1-2 additional fractions if they have the positive surgical margin. Treatment tolerance was assessed and considered satisfactory. Long-term toxicity was greater. Such toxicity was acceptable taking into consideration the fact that it rarely seems to impact patients' quality of life, as most cases have been reported as asymptomatic along the follow-up. The results regarding specific survival and local control were similar to the standard treatment and matched with the series already published, stressing that the survival of these older patients is more influenced by their own comorbidities than for the evolution of breast cancer, leading to a specific survival of 90% at 5 years.

In addition, in our series, we have irradiated the supraclavicular fossa (in 15% of the series) in those patients with involvement of 4 or more axillary lymph nodes, in patients with 1–3 involved nodes during the period from 2008 to 2016, those with insufficient lymphadenectomy (less than 10–12 nodes), or those with macroscopically supraclavicular involvement. The regional treatment volume was on the nodes of the supraclavicular fossa. Radio dermatitis is the most common acute toxicity and leads to the most discomfort for patients [29]. Signs and symptoms that are derivate from breast irradiation are conditioned by various factors, such as the volume to be treated, the irradiation technique, and the energy of the fractionation, the total dose, as well as previous and/or concomitant systemic treatments and comorbidities of the patient [30]. Radio induced skin toxicity has been extensively documented and investigated, but in most cases there is no standardization in prevention and care [31, 32]. The chronic effects of irradiation at the cutaneous level include hyperpigmentation, telangiectasia, subcutaneous fibrosis, edema, and local pain that may continue to occur in the subsequent and long-term follow-up of irradiated patients [33]. In our series, although acute and chronic toxicity was acceptable and seems not to compromise quality of life in most cases, the percentage of patients with fibrosis increased over time, which presented 30.7% at a median follow-up of 51 months. Agrawal et al. compared the standard schedule (2 Gy × 25 s) with the weekly hypofraction scheme in 5.7 Gy × 5 s or 6 Gy × 5 and after a 3-year follow-up showed that 28.5 Gy or 30 Gy in 5 sessions are comparable to 50 Gy in 25 sessions. The 28.5 Gy schedule had a slightly lower acute toxicity and a more discreet cosmetic impairment [34].

Subsequently, the FAST Forward trial that compares the daily schedule in one week with the UK standard schedule at 3 weeks after primary surgery for early breast cancer is ongoing. With a moderate reduction of the dose, it seems that there is relatively better tolerance and less acute toxicity. However, longer follow-up is required [35].

We decided to apply the 5 Gy scheme in 6 sessions instead of the 6.25 Gy × 6 schedule starting in August 2012, with the intention of reducing chronic toxicity without compromising the oncologic result. Therefore, we included 45 patients (9.3%) in the present study in which we administered 5 Gy per fraction. No difference in survival has been shown. Although not significant, a trend towards lower chronic toxicity with 5 Gy has been observed in comparison with the 6.25 Gy schedule in 6 sessions, although further monitoring is necessary to confirm this data. In addition, in the last years there was a tendency to include the patients with worse physical conditions or comorbidities in such weekly schedule. We are looking forward to obtain good clinical results and lower toxicity with a longer follow-up.

We irradiated whole breast after conservative surgery, but actually there is a consolidated experience in schedules in which the partial irradiation of the breast in selected favorable cases is considered to be a more comfortable alternative for the patients. It is possible to administer a high enough dose to the involved quadrant of the breast. The most frequent schedules consist in 3.85 Gy doses twice a day, ten fractions in a week, although other schedules have been utilized. Also the partial breast irradiation can be performed by a single intraoperative session [36]. This treatment achieves similar results in terms of toxicity and local control, constituting a good option in low risk patients [37]. Also, actually there are several studies ongoing investigating the omission of the adjuvant irradiation in elderly good prognosis patients after conservative surgery.

## 5. Conclusions

Weekly hypofractionated radiation therapy is a feasible and convenient option for patients with advanced breast cancer who do not tolerate a daily treatment. It is a safe treatment modality with similar survival and local control results. The side effects are also acceptable. As with the weekly hypofractionation schedule, the reduction of total time treatment may improve patients' quality of life.

## Figures and Tables

**Table 1:** Characteristics of the patients.

	*n*	%
*Stage*		
I	187	38.5
II	182	37.4
III	80	16.5
IV	5	1
In situ	11	2.26
Recurrence	10	2
Unknown	11	2.3

*Histologic grade*		
1	85	17.5
2	218	44.8
3	100	20.5
Unknown	83	17

*Surgical type*		
Brest-conserving surgery	382	78.6
Mastectomy	97	20

**Table:** (a) Daily hypofractionated schedules

Author	*n*	RDT schedule	Commentary
Shelley et al., 2000 [15]	294	40 Gy in 2.5 Gy/s, 22 days	

Deutsch, 2002 [38]	47	Different schedules since 50 Gy in 2 Gy to 36 Gy in 3 Gy	80–89 years old

Whelan et al., 2002 [39]	1234	Standard schedule 50 Gy in 2 Gy/s, 35 days, versus hypofraction 42.5 Gy in 2.65 Gy/s, 22 days	

Yarnold et al., 2005 [37]	1410	50 Gy in 2 Gy/s, 39 Gy in 3 Gy/s, or 42.9 Gy in 3.3 Gy/s	

Haviland et al., 2013 [28]	2236	START A: 50 Gy in 2 Gy/s versus 41.6 Gy or 39 Gy in 13 s START B: 50 Gy in 2 Gy/s versus 40 Gy in 15 s	

**Table:** (b) Weekly hypofractionated schedules

Author	*n*	RDT schedule	Commentary
Rostom et al., 1987 [21]	**84**	6.5 Gy × 6 s	

Bates, 1988 [40]	411	3 Gy × 12 s (3 s/week = 28 days) versus 6 Gy × 6 s (2 s/week = 18 days)	53 p biopsy, 13 p lumpectomy, 18 p mastectomy

Baillet et al., 1990 [41]	230	45 Gy (1.8 Gy × 25 s in 33 days) versus 23 Gy (5.75 Gy × 4 s in 17 days)	

Maher et al., 1995 [23]	70	6.5 Gy × 5 s + boost 6.5 Gy × 2 s	Biopsy or cytology only

Ortholan et al., 2005 [22]	150	6.5 Gy × 5 s; supra 5.5 Gy × 5 s; boost 6.5 Gy × 1 or 2 s	108 p lumpectomy, 43 p mastectomy

Courdi et al., 2006 [24]	115	6.5 × 5 s	Median age 83 years, biopsy only

Sanz et al., 2008 [7]	262	6.25 Gy × 6 s (+6.25 Gy × 2 s if positive margin in conservative surgery)	22 p biopsy, 174 p lumpectomy, 66 p mastectomy

Dragun et al., 2011 [26]	42	6 Gy × 5 s	69% lumpectomy 31% chemotherapy

Yarnold, 2011 [25]	915	50 Gy (2 Gy × 25 s) versus 28.5 Gy (5.7 × 5 s) or 30 Gy (6 Gy × 5 s)	

Rovea et al., 2015 [27]	298	30 Gy (6 Gy × 5 s) or 32.5 Gy (6.5 Gy × 5 s)	

Brunt et al., 2016 [35]	352	40 Gy in 15 s in 3 weeks versus 27 Gy in 5 s in 1 week or 26 Gy in 5 s in 1 week	Patients that require lymph node irradiation are excluded

RDT: radiation therapy; s: session; p: patient.
